# Comparison of hyperreflective foci in macular edema secondary to multiple etiologies with spectral-domain optical coherence tomography: An observational study

**DOI:** 10.1186/s12886-022-02575-9

**Published:** 2022-08-29

**Authors:** Ruilin Zhu, Shiyu Xiao, Wenbo Zhang, Jun Li, Menglu Yang, Yadi Zhang, Xiaopeng Gu, Liu Yang

**Affiliations:** 1 Department of Ophthalmology, Peking University First Hospital, Beijing, 100034 China; 2 Department of Ophthalmology, Schepens Eye Research Institute, Massachusetts Eye and Ear, Harvard Medical School, Boston, MA 02114 USA

**Keywords:** Macular edema, Hyperreflective foci, Spectral-domain optical coherence tomography, Uveitis

## Abstract

**Background:**

Hyperreflective foci (HRF) features in macular edema associated with different etiologies may indicate the disease pathogenesis and help to choose proper treatment. The goal of this study is to investigate the retinal microstructural features of macular edema (ME) secondary to multiple etiologies with spectral-domain optical coherence tomography (SD-OCT) and analyze the origin of HRF in ME.

**Methods:**

This was a retrospective study. SD-OCT images were reviewed to investigate macular microstructural features such as the number and distribution of HRF and hard exudates and the internal reflectivity of the cysts. The differences in microstructural features between groups and the correlations between the number of HRF and other parameters were analyzed.

**Results:**

A total of 101 eyes with ME from 86 diabetic (diabetic macular edema, DME) patients, 51 eyes from 51 patients with ME secondary to branch retinal vein occlusion (branch retinal vein occlusion-macular edema, BRVO-ME), 59 eyes from 58 central retinal vein occlusion (central retinal vein occlusion-macular edema, CRVO-ME) patients, and 26 eyes from 22 uveitis (uveitic macular edema, UME) patients were included in this study. The number of HRF, the frequency of hard exudates and the enhanced internal reflectivity of the cysts were significantly different among the groups. The number of HRF in the DME group was significantly higher than that in the other groups (all *P* < 0.05). The frequency of hard exudates and enhanced internal reflectivity of the cysts in the DME group were significantly higher than ME secondary to other etiologies (all *P* < 0.001). Within the DME group, the number of HRF in the patients with hard exudates was significantly higher than that in the patients without hard exudates (*P* < 0.001).

**Conclusion:**

HRF detected with SD-OCT were more frequent in DME patients than in BRVO-ME, CRVO-ME, or UME patients. The occurrence of HRF was correlated with the frequency of hard exudates. HRF may result from the deposition of macromolecular exudates in the retina, which is speculated to be a precursor of hard exudates.

## Background

Macular edema (ME) is a major cause of visual impairment in a variety of diseases including metabolic, vascular, and inflammatory retinal diseases [1–3]. As reported, macular edema is present in over 20% of diabetic patients 20 years after diagnosis [4], in 5%–15% of retinal vein occlusion patients [5], and 20%–35% of uveitis patients [6]. Evidence show that both the pro-inflammatory mediators and vascular endothelial growth factor (VEGF) are the leading causes of macular edema [7], thus ME can be controlled with anti-VEGF or anti-inflammatory agents. However, clinically patients respond inconsistently to these treatments. Choosing the proper therapeutic method specifically for each individual remains a challenge in clinical practice. Finding an appropriate marker indicating the difference of ME among the patients is crucial not only for better understanding the pathogenesis of the disease, but also valuable for choosing the most suitable treatment for the patients.

Spectral-domain optical coherence tomography (SD-OCT) is a noninvasive retinal imaging technique, which is currently widely used in retinal diseases, including macular edema. SD-OCT provides high-resolution retinal microstructure images, which greatly facilitate the diagnosis and follow-up of macular edema [8]. Typically, ME presents as hyporeflective cystoid spaces on SD-OCT scans. In some patients however, hyperreflective dots can be observed scattered throughout all retinal layers, primarily around the intraretinal cystoid spaces, commonly known as hyperreflective foci (HRF) [9, 10]. The presence of HRF have been found in macular edema associated with diabetes, retinal vein occlusion, uveitis, age-related macular degeneration, and other diseases [11–16]. However, the pathogenesis of HRF have not been fully elucidated. Researchers have hypothesized that the development of HRF is the precursor to hard exudates [12, 17, 18] or the activation of inflammatory cells [19–22]. It is believed that the presence of HRF may be important for treatment regimen, and the number of HRF indicate the prognosis of macular edema [19, 23–26]. However, it remains unclear whether the number of HRF differs among multiple etiologies of macular edema.

Clinically, another newly described SD-OCT feature is believed to be correlated with HRF, which is enhanced internal reflectivity of the edema cyst [27, 28]. The enhanced internal reflectivity of the cyst was defined as the presence of clump of hyperreflective material present in the intraretinal space [28] or presence of a plume-shaped hyper-reflective substance within the cyst [29]. In addition to the correlation with HRF, the enhanced internal reflectivity of the cyst is also believed to be related to ME prognosis [27], thus the enhanced internal reflectivity is also described along with HRF in ME patients.

In this study, we compared the HRF characteristics among several leading etiologies of macular edema, including diabetic retinopathy (DR), branch retinal vein occlusion (BRVO), central retinal vein occlusion (CRVO), and uveitis, analyzed the correlation between the number of HRF and hard exudates. Along with HRF, the enhanced internal reflectivity of the cyst was also investigated. Additionally, the correlations between HRF and the features of hard exudates in DME patients was studied to analyze the properties and origin of HRF.

## Methods

### Subjects

This study was approved by the Institutional Review Board of Peking University First Hospital. Medical records of diabetic macular edema (DME), macular edema secondary to branch retinal vein occlusion (branch retinal vein occlusion-macular edema, BRVO-ME), macular edema secondary to central retinal vein occlusion (central retinal vein occlusion-macular edema, CRVO-ME), and noninfectious uveitic macular edema (UME) patients diagnosed at the Department of Ophthalmology, Peking University First Hospital between January 2015 and October 2019 were reviewed retrospectively. The SD-OCT images of each patient were reviewed. Both eyes of the same patient were included if the ME was bilateral and shared the same etiology.

The exclusion criteria were as follows:

- 1) Low SD-OCT image quality due to unclear refracting media.
- 2) Intravitreal anti-vascular endothelial growth factor injection within 3 months prior to the SD-OCT image.
- 3) Cataract surgery or refractive surgery within 3 months prior to the SD-OCT image.
- 4) Dexamethasone intravitreal implant or fluocinolone acetonide intravitreal implant within 6 months prior to the SD-OCT image; Systemic or periocular corticoid treatment within 6 months prior to the SD-OCT image.
- 5) Comorbidity with other fundus diseases, glaucoma, vitreous macular traction, pathologic myopia, or a history of vitrectomy or fundus laser treatment.
- 6) Presence of proliferative diabetic retinopathy.
- 7) UME due to Vogt-Koyanagi-Harada disease.

### SD-OCT Imaging

The SD-OCT images (Heidelberg Engineering, Heidelberg, Germany) from all study groups were obtained with a horizontal scan centered on the fovea using an enhanced depth imaging (EDI) protocol and 50 automated real-time repetitions. The retinal layers were termed the inner retinal layer (IRL, comprising the layers between the inner limiting membrane (ILM) and the inner nuclear layer (INL)), middle retinal layer (MRL, comprising the outer plexiform layer (OPL) and the outer nuclear layer (ONL)), and outer retinal layer (ORL, comprising the layers between the external limiting membrane (ELM) and the retinal pigment epithelium (RPE)).

SD-OCT images were analyzed by two experienced ophthalmologists (RZ and SX). They were masked to all clinical information including diagnosis, and consistency between the two researchers was tested. A third experienced ophthalmologist was invited to make decisions in case of disagreement to reduce relevant errors (WZ). The following morphological characteristics were recorded:

- 1) Hard exudates (hyperreflective signal with an irregular shape in the retina, accompanied by an obvious posterior shadow, corresponding to the yellow or high-density lesions visible in the color or infrared fundus image).
- 2) Enhanced internal reflectivity of the cyst (the hyperreflective signal of the contents in the retinal edema cyst, which was significantly stronger than that of the vitreous reflectivity).

Based on previous reports [24, 30], HRF were defined as discrete and dotted signals in the retina with clear boundaries, with diameters of 10-40 μm, to avoid missing smaller HRFs and exclude hard exudate. Their reflective signals were defined as higher than or equal to that of the RPE to rule out signal noise. Hyperreflective signals were not considered HRF if the signals aggregated and the corresponding lesions could be seen on the color fundus or infrared images. The numbers of HRF were counted in different retinal layers.

### Statistical analysis

The statistical software SPSS 24.0 (IBM, SPSS Statistics) was used for data analysis. Classified variables were expressed as frequencies (n) and percentages (%); non-normally distributed continuous variables were expressed as quaternary *M (P25, P75)*. *P* < 0.05 was considered to indicate a statistically significant difference, and the corresponding Bonferroni correction was performed for statistical significance values during intragroup paired comparisons after multigroup comparisons.

- 1) The overall differences in sex, hard exudate frequency, and enhanced internal reflectivity of the cysts among groups were statistically analyzed with the chi-square test or Fisher's exact test. The DME group was compared with the other groups using Kruskal–Wallis test, and the corresponding Bonferroni correction was performed for the statistical significance value.
- 2) After the normality and homogeneity of variance tests were conducted on the numbers of HRF among the groups, the differences in the numbers of HRF between the overall groups were statistically analyzed by independent-sample Kruskal–Wallis test, and then pairwise comparisons were made between the DME group and the other groups. The corresponding Bonferroni correction was performed for the statistical significance value.
- 3) The DME group was divided into two subgroups according to the frequency of hard exudates and the enhanced internal reflectivity of the cyst. The number of HRF in each retinal layer in the DME group was compared between the two subgroups. Two independent-sample Mann–Whitney U tests were used for statistical analysis.

## Results

Two hundred thirty-seven eyes from 217 patients were included in this study; 101 eyes were from 86 DME patients, 51 from 51 BRVO-ME patients, 59 from 58 CRVO-ME patients, and 26 from 22 UME patients. The demographic characteristics of each group are shown in Table 1. The overall distribution of age and sex among the groups was significantly different, with *P* < 0.001and *P* = 0.009, respectively.

**Table 1:** Demographic characteristics of the participants

	DME (*n* = 101)	BRVO-ME (*n* = 51)	CRVO-ME (*n* = 59)	UME (*n* = 26)	*χ*^2^	*P*
Age, years *M (P25, P75)*	60.00 (55.00, 66.00)	58.00 (52.00, 68.00)	60.00 (51.00, 70.00)	50.00 (36.00, 56.25)	-	< 0.001***
Male, n (%)	71 (70.3%)	23 (45.1%)	40 (67.8%)	13 (50.0%)	11.569	0.009**
Right eye, n. (%)	59 (58.4)	29 (56.9%)	35 (59.3%)	15 (57.7%)	0.073	0.995

*DME* Diabetic macular edema, *BRVO-ME* Branch retinal vein occlusion-macular edema, *CRVO-ME* Central retinal vein occlusion-macular edema, *UME* Uveitic macular edema. *M (P25, P75)* Medium and quartile

^**^P < 0.01

^***^P < 0.001

### Hard exudates and enhanced internal reflectivity are observed more frequently in eyes with DME

**Fig. 1:**
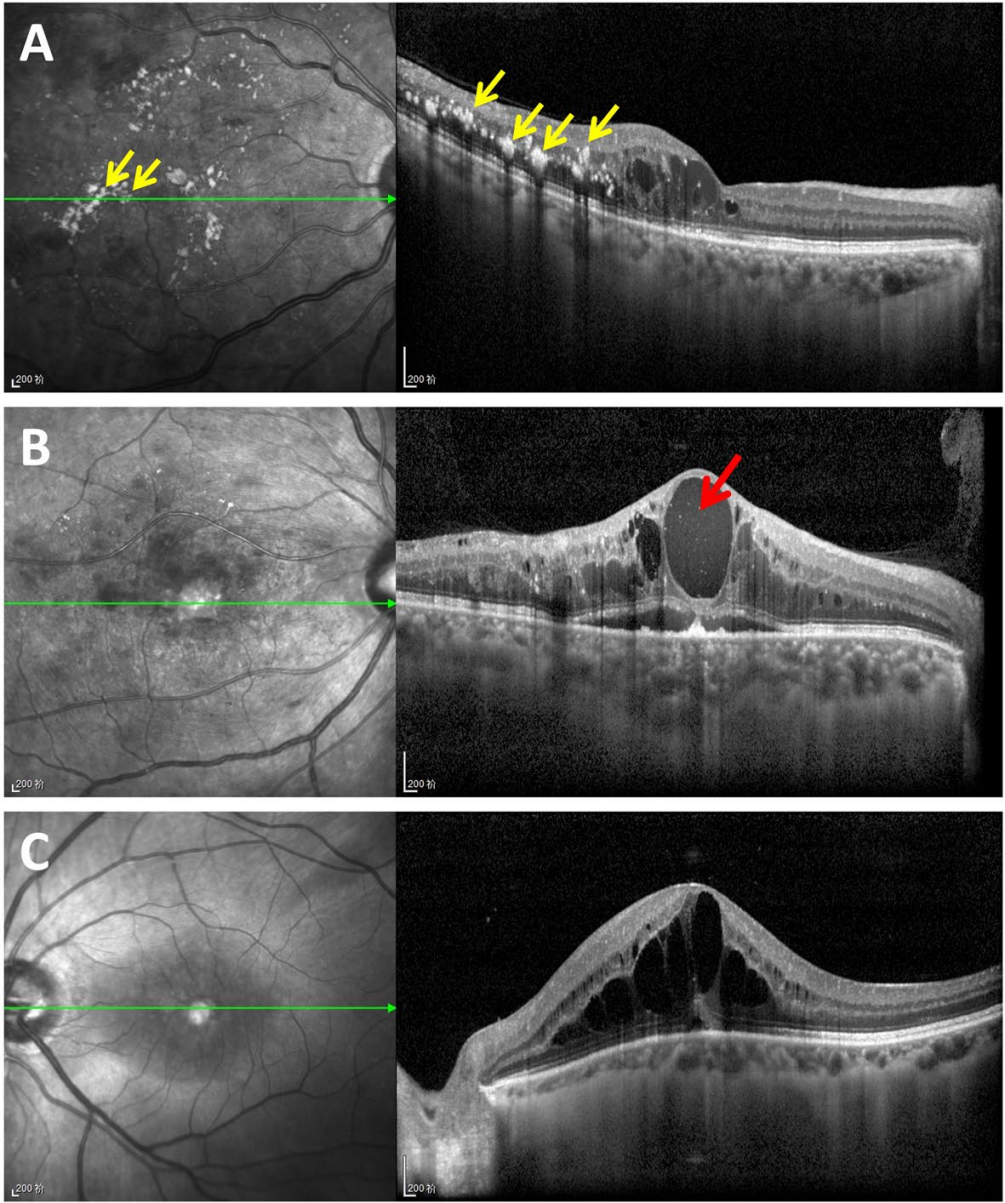
Representative SD-OCT images of macular edema secondary to different etiologies. **A** Diabetic macular edema (DME), hard exudates are seen in the retina (yellow arrows), manifesting as irregular dense and hyperreflective signals, mainly distributed in the outer plexiform layer, accompanied by a posterior hyporeflective shadow, and corresponding exudate lesions can be seen in infrared fundus images. **B** DME, the signal in the edema cyst was enhanced (red arrow), showing that the reflectivity in the edema cyst was higher than that in the vitreous. **C** Uveitic macular edema (UME), no obvious hard exudates or enhanced internal reflectivity of the cyst was observed, no high-density lesion was observed in the corresponding infrared fundus images, and the reflectivity of the contents in the cyst was equivalent to that in the vitreous

**Fig. 2:**
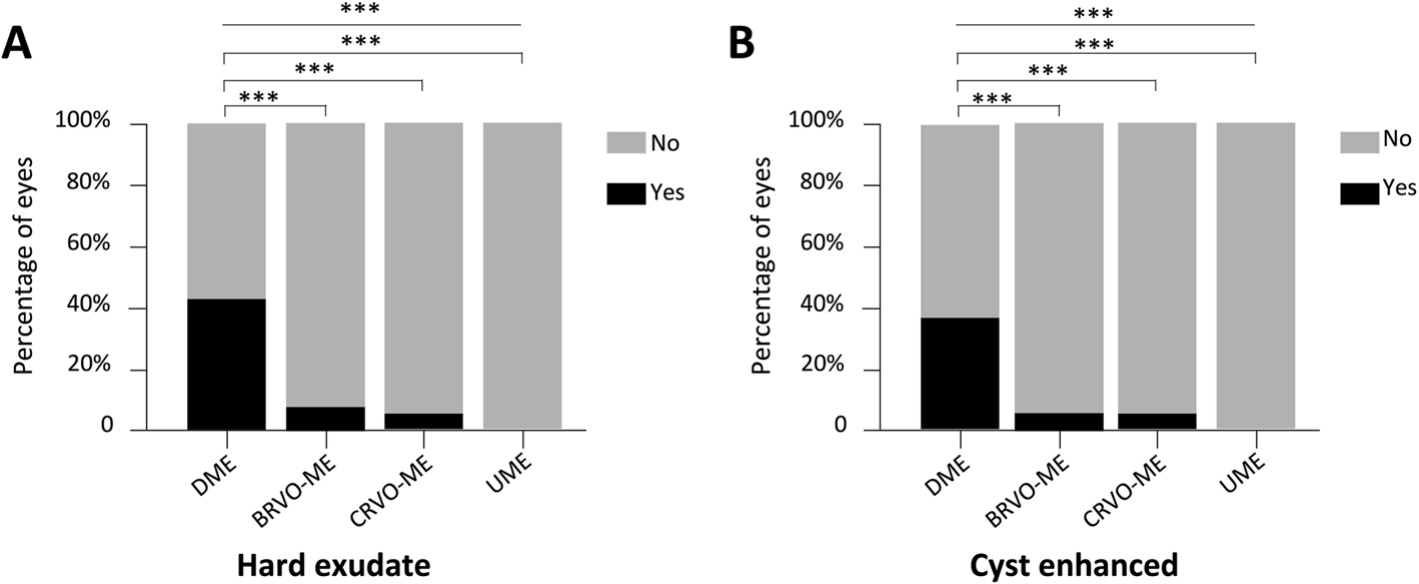
The frequency of hard exudates and enhanced internal reflectivity of the cyst in different groups. **A** Hard exudates; (**B**) Enhanced internal reflectivity of cyst. DME: diabetic macular edema, BRVO-ME: branch retinal vein occlusion-macular edema, CRVO-ME: central retinal vein occlusion-macular edema, UME: uveitic macular edema. M (P25, P75): medium and quartile.*** indicates *P* < 0.001

Hard exudates and enhanced internal reflectivity of the cysts were more frequently seen in the DME patients. The frequency of hard exudates in the DME, BRVO-ME, CRVO-ME and UME patients were 42.6%, 7.8%, 5.1% and 0, respectively. The result was significantly different among the groups (*P* < 0.001). The occurrence rates of the enhanced internal reflectivity of the cyst in the four groups were 36.6%, 5.9%, 5.1% and 0, respectively. In DME group, the enhanced internal reflectivity of the cysts presented more frequently than in other groups and the difference was significant (*P* < 0.001). The frequency of hard exudates and enhanced internal reflectivity of the cysts in macular edema of different etiologies are shown in Figs. 1, 2 and Table 2.

**Table 2:** The frequency of hard exudates and enhanced internal reflectivity of the cyst in different groups

	DME *(n* = 101)	BRVO-ME (*n* = 51)		CRVO-ME (*n* = 59)	UME (*n* = 26)	*χ*^2^	*P*
Hard exudates + n (%)	43 (42.6%)	4 (7.8%)		3 (5.1%)	0 (0.0%)	49.409	< 0.001***
# DME vs. BRVO-ME		-	-		-	19.138	< 0.001***
# DME vs. CRVO-ME		-	-		-	25.553	< 0.001***
# DME vs. UME		-	-		-	16.736	< 0.001***
Enhanced internal reflectivity of the cyst + n (%)	37 (36.6%)	3 (5.9%)	3 (5.1%)		0 (0.0%)	40.950	< 0.001***
# DME vs. BRVO-ME		-	-		-	16.527	< 0.001***
# DME vs. CRVO-ME		-	-		-	19.771	< 0.001***
# DME vs. UME		-	-		-	13.440	< 0.001***

*DME* Diabetic macular edema, *BRVO-ME* Branch retinal vein occlusion-macular edema, *CRVO-ME* Central retinal vein occlusion-macular edema, *UME* Uveitic macular edema

^***^P < 0.001

### HRF are observed more frequently in eyes with DME

**Fig. 3:**
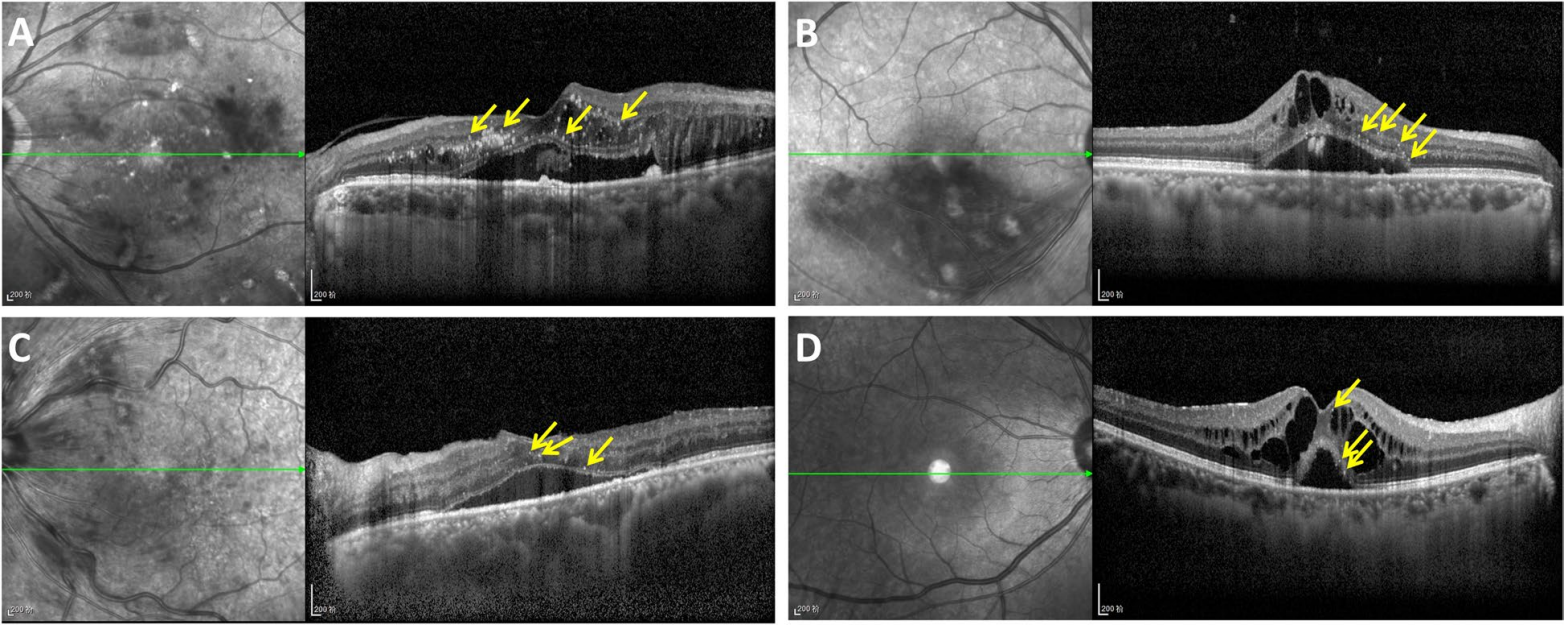
The representative SD-OCT images of macular edema showing HRF in different diseases. The HRF can be seen in the retinas (yellow arrows) (**A**) Diabetic macular edema (DME). **B** Branch retinal vein occlusion-macular edema (BRVO-ME). **C** Central retinal vein occlusion-macular edema (CRVO-ME). **D** Uveitic macular edema (UME)

The number of HRF in different retinal layers were analyzed. Representative images of HRF were shown in Fig. 3. HRF were more frequently observed in the DME group in each retinal layer compared with other groups. In the full-layer retina (ILM-RPE), the mean number of HRF were 14 in DME group, 9 in BRVO-ME group, 8 in CRVO-ME group, and 6 in the UME group. The difference was significant among the groups (*P* < 0.001).

**Fig. 4:**
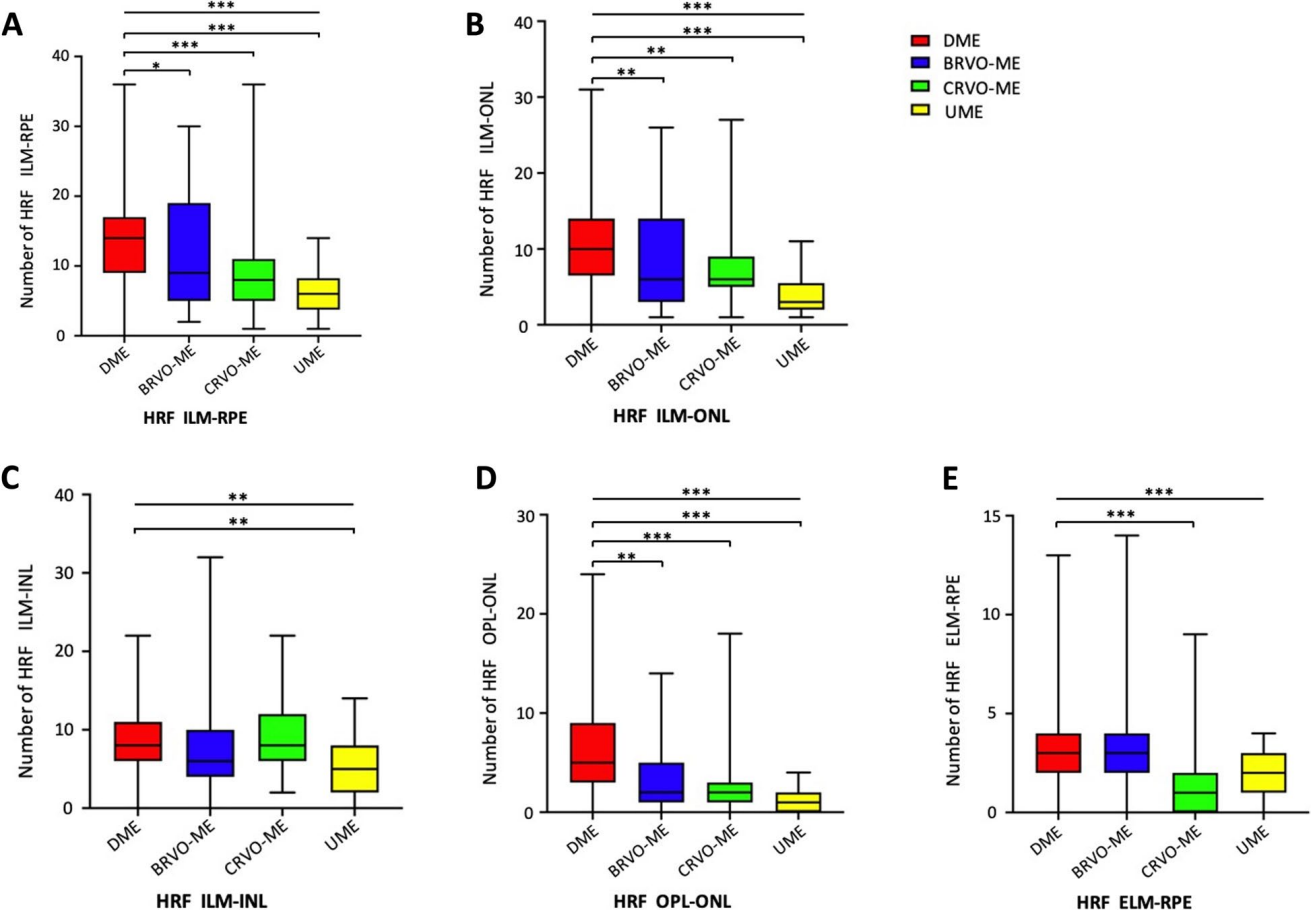
The number of hyperreflective foci (HRF) in different retinal layers. **A** ILM-RPE layer; **B** ILM-ONL; **C** ILM-INL; **D** OPL-ONL; (E) ELM-RPE layer. *P < 0.05, **P < 0.01, ***P < 0.001. ILM: inner limiting membrane, RPE: retinal pigment epithelium, ONL: outer nuclear layer, INL: inner nuclear layer, OPL: outer plexiform layer, ELM: external limiting membrane. DME: diabetic macular edema, BRVO-ME: branch retinal vein occlusion-macular edema, CRVO-ME: central retinal vein occlusion-macular edema, UME: uveitic macular edema

The number of HRF in the DME group in ILM-ONL, ILM-INL, OPL-ONL and ELM-RPE layer were shown in Table 3 and Fig. 4. In ILM-ONL and OPL-ONL layer, the number of HRF in the DME group was significantly higher than that in the other groups (*P* < 0.01). In ILM-INL layer, HRF in the DME group was significantly more frequent than that in the UME group (*P* = 0.009). No significant difference was found between the DME group and the BRVO-ME (*P* = 0.606), nor between the DME and CRVO-ME group (*P* = 1.00) in this layer. In the ELM-RPE layer, HRF amount in the DME group was significantly higher than that in the CRVO-ME group (*P* < 0.001), but when compared the DME group with BRVO-ME and UME group, there was no significant difference (*P* = 1.00, *P* = 0.218). No significant difference in HRF numbers was found between males and females or among the age groups (all *P* > 0.05).

**Table 3:** The number of HRF in different retinal layers of macular edema

	DME (*n* = 101)	BRVO-ME (*n* = 51)	CRVO-ME (*n* = 59)	UME (*n* = 26)	Z	*P*
ILM-RPE	14.00 (9.00, 17.00)	9.00 (5.00, 19.00)	8.00 (5.00, 11.00)	6.00 (3.75, 8.25)	-	< 0.001***
# DME vs. BRVO-ME		-	-	-	2.682	0.044*
# DME vs. CRVO-ME		-	-	-	4.866	< 0.001***
# DME vs. UME		-	-	-	5.549	< 0.001***
ILM-ONL	10.00 (6.50, 14.00)	6.00 (3.00, 14.00)	6.00 (5.00, 9.00)	3.00 (2.00, 5.00)	-	< 0.001***
# DME vs. BRVO-ME		-	-	-	3.367	0.005**
# DME vs. CRVO-ME		-	-	-	3.497	0.003**
# DME vs. UME		-	-	-	5.969	< 0.001***
ILM-INL	4.00 (3.00, 5.00)	3.00 (2.00, 5.00)	4.00 (3.00, 6.00)	2.50 (1.00, 4.00)	-	0.001**
# DME vs. BRVO-ME		-	-	-	1.640	0.606
# DME vs. CRVO-ME		-	-	-	-1.148	1.000
# DME vs. UME		-	-	-	3.187	0.009**
OPL-ONL	5.00 (3.00, 9.00)	2.00 (1.00, 5.00)	2.00 (1.00, 3.00)	1.00 (0.00, 2.00)	-	< 0.001***
# DME vs. BRVO-ME		-	-	-	3.863	0.001**
# DME vs. CRVO-ME		-	-	-	5.512	< 0.001***
# DME vs. UME		-	-	-	6.288	< 0.001***
ELM-RPE	3.00 (2.00, 4.00)	3.00 (2.00, 4.00)	1.00 (0.00, 2.00)	2.00 (1.00, 3.00)	-	< 0.001***
# DME vs. BRVO-ME		-	-	-	-0.530	1.000
# DME vs. CRVO-ME		-	-	-	6.332	< 0.001***
# DME vs. UME		-	-	-	2.093	0.218

The number of HRF is denoted by medium and quartile *M* (*P*_*25*_*, P*_*75*_).*HRF* Hyperreflective foci, *DME* Diabetic macular edema, *BRVO-ME* Branch retinal vein occlusion-macular edema, *CRVO-ME* Central retinal vein occlusion-macular edema, *UME* Uveitic macular edema

^*^P < 0.05

^**^P < 0.01

^***^P < 0.001

### More HRFs were observed in DME with hard exudate

**Fig. 5:**
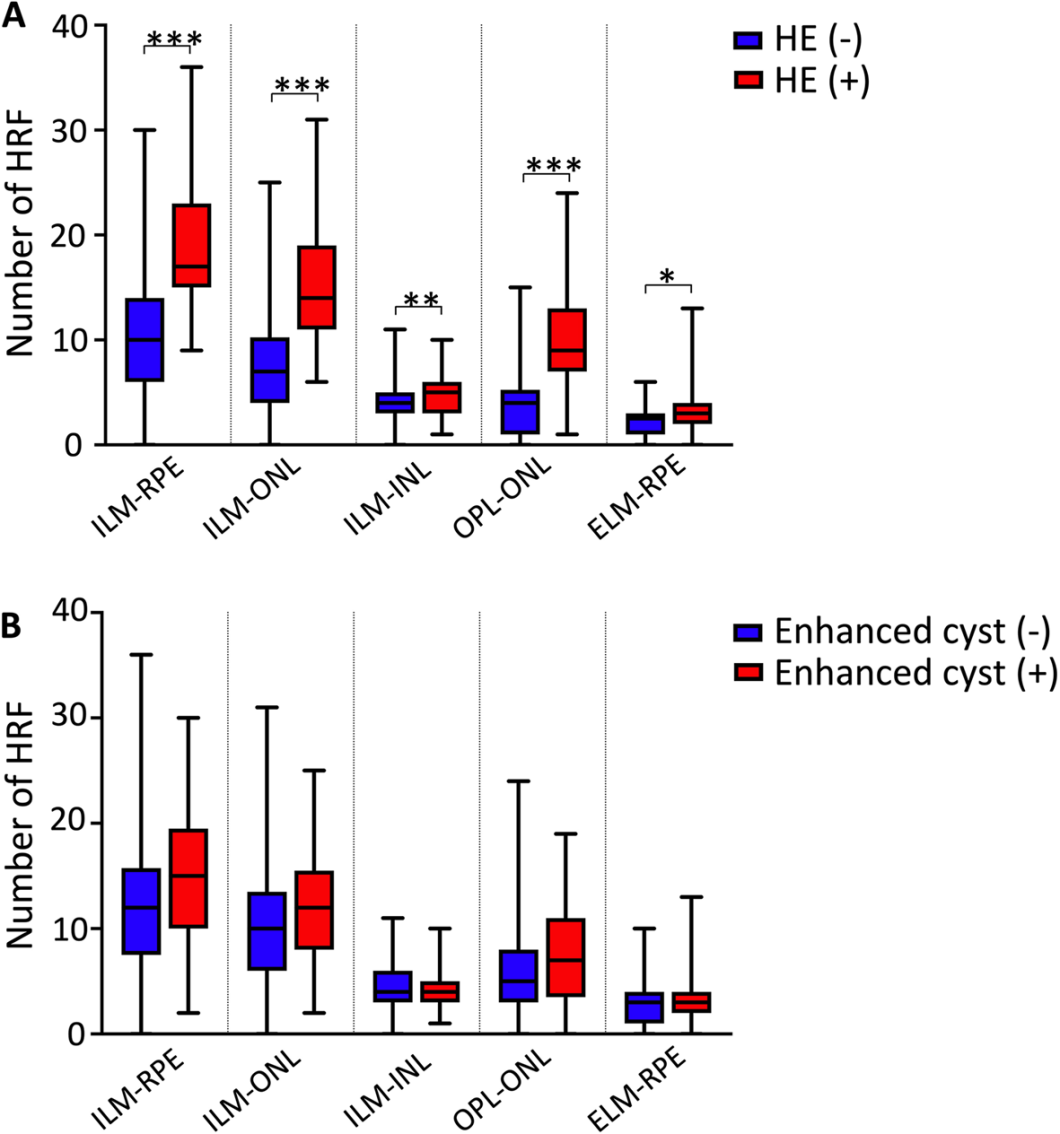
Hyperreflective foci (HRF) and hard exudates and enhanced internal reflectivity of the cyst in diabetic macular edema (DME) patients. **A** The number of HRF in each retinal layer between the hard exudate subgroups. **B** The number of HRF in each retinal layer between the subgroups based on enhanced internal reflectivity of the cyst. HE: Hard exudates. *P < 0.05, **P < 0.01, ***P < 0.001

The DME patients were divided into two subgroups according to the presence of hard exudates in the retina. The correlation of HRF and hard exudates were analyzed. The number of HRF in each retinal layer was significantly greater in the hard exudate ( +) subgroup (all *P* < 0.05). The results were shown in Fig. 5 (A) and Table 4.

**Table 4:** The number of HRF in each retinal layer between the hard exudate subgroups

	Hard exudates		*t*/*Z*	*P*
	No (*n* = 58)	Yes (*n* = 43)		
ILM-RPE	10.34 ± 5.243	18.56 ± 6.196	-7.202	< 0.001***
ILM-ONL	7.86 ± 4.351	14.93 ± 5.184	-7.438	< 0.001***
ILM-INL	4.00 (3.00, 5.00)	5.00 (3.00, 6.00)	2.694	0.007**
OPL-ONL	4.00 (1.00, 5.25)	9.00 (7.00, 13.00)	6.503	< 0.001***
ELM-RPE	2.50 (1.00, 3.00)	3.00 (2.00, 4.00)	2.313	0.021*

*HRF* Hyperreflective foci, *ILM* Inner limiting membrane, *RPE* Retinal pigment epithelium, *ONL* Outer nuclear layer, *INL* Inner nuclear layer, *OPL* Outer plexiform layer, *ELM* External limiting membrane. The number of HRF is denoted by mean ± SD or medium and quartile *M* (*P*_*25*_*, P*_*75*_)

^*^P < 0.05,

^**^P < 0.01,

^***^P < 0.001

### No association between the number of HRF and the presence of enhanced internal reflectivity in eyes with DME

The presence of enhanced internal reflectivity of the cysts in the retina was analyzed within the DME group. No significant difference was shown in the number of HRF in each layer between eyes with or without enhanced internal reflectivity of the cyst in DME group (Table 5 and Fig. 5 (B)).

**Table 5:** The number of HRF in each retinal layer between the enhanced internal reflectivity of the cyst subgroups

	Enhanced internal reflectivity of the cyst		*t*/*Z*	*P*
	No (*n* = 64)	Yes (*n* = 37)		
ILM-RPE	12.00 (7.50, 15.75)	15.00 (10.00, 19.50)	1.825	0.068
ILM-ONL	10.00 (6.00, 13.50)	12.00 (8.00, 15.50)	1.674	0.094
ILM-INL	4.00 (3.00, 6.00)	4.00 (3.00, 5.00)	1.329	0.184
OPL-ONL	5.00 (3.00, 8.00)	7.00 (3.50, 11.00)	1.432	0.152
ELM-RPE	3.00 (1.00, 4.00)	3.00 (2.00, 4.00)	0.927	0.354

*HRF* Hyperreflective foci, *ILM* Inner limiting membrane, *RPE* Retinal pigment epithelium, *ONL* Outer nuclear layer, *INL* Inner nuclear layer, *OPL* Outer plexiform layer, *ELM* External limiting membrane. The number of HRF is denoted by medium and quartile *M* (*P*_*25*_*, P*_*75*_)

## Discussion

In this investigation, we analyzed SD-OCT images acquired from patients with macular edema secondary to DR, BRVO, CRVO, and noninfectious uveitis. Our results show that the frequency of hard exudates and enhanced reflectivity in edema cysts in the DME group was significantly higher than that in BRVO-ME, CRVO-ME and UME eyes. The HRF number in DME eyes were significantly higher than that in the other groups, while the lowest HRF number was observed in eyes with UME. In eyes with DME, the number of HRF in each retinal layer is associated with the presentation of hard exudate.

The mechanism behind macular edema due to each etiology varies. In RVO, macular edema is believed to be raised from venous reflux disorder, increased capillary hydrostatic pressure, and blood-retinal barrier (BRB) breakdown caused by tissue ischemia and hypoxia [31]; while in uveitis, the ME is due to the leaky BRB and increased osmotic pressure resulted from inflammation [1]. The pathogenesis of DME is a combination of multiple factors including the increase of inflammatory condition, alteration of vascular structure, breakdown of BRB, degeneration of Müller cells [4, 7, 32]. Dysli et al. [32] suggested different characteristic SD-OCT patterns are often indicative of the underlying cause and pathology of macular edema. Hecht et al. [33] differentiated DME from pseudophakic cystoid macular edema with machine learning algorithm based on the SD-OCT characteristics. HRF was one of the important markers [32, 33]. The different characteristics of HRF among the diseases may reflect the pathogenesis of macular edema secondary to multiple etiologies. Identifying the origin of HRF may contribute to optimize treatment options. Munk et al. [34] reported 100% CRVO-ME patients, 98% of DME patients, 65% of BRVO-ME patients and 0% of UME patients had HRF. Our investigation showed that the number of HRF was the highest in the DME group compared to the BRVO-ME, CRVO-ME, and UME groups. The difference with CRVO-ME may be caused by sampling difference.

The origin of HRF has drawn the interests of numerous teams around the world. Some studies [12, 17, 18] supported that HRF in DME are precursors of hard exudates, representing the exudation of macromolecular substances such as proteins and lipids after BRB destruction; other groups [13, 19, 21, 35] believed the hyperreflective dots represent an inflammatory response, which originated from microglial cells or white blood cells infiltration. In the current findings, the number of HRF is the lowest in UME, while the associated with hard exudates further supports the hypothesis that HRF are precursors of hard exudates, rather than the product of inflammatory activation.

Noticeably, in studies with positive associations between HRF and inflammatory conditions, the hyperreflective dots observed on SD-OCT images were smaller in size and lower in reflective strength [21]. The signal intensity of the HRF is reported to be lower than that of the RPE layer and closer to the retinal nerve fiber layer (RNFL) [13, 19, 35]. Vujosevic et al. [30] reported that in DME SD-OCT images, HRF with a larger diameter and signal intensity similar to that of the RPE may be a precursor of hard exudates, while HRF with a smaller diameter and signal intensity similar to that of the RNFL may represent microglia. Moreover, when the reflectivity is lower than that of the RPE layer or approximately equal to that of the RNFL, signal noise or blood vessels in the retina might be misjudged as HRF. Therefore, the discrepancy of the definition of HRF by different groups is an important reason for inconsistent results, and the interpretation standards of HRF needs to be defined more carefully. In the current study, the signal intensity of HRF was defined as the same as RPE layer, and the minimum diameter of HRF was set to 10 μm to avoid miss counting smaller HRFs. Even though the counting of HRF were done carefully, certain bias is still inevitable as mentioned in other reports [14, 19, 24, 35–37], for example, the selected section of analysis was limited to the central horizontal SD-OCT B-scan. In the future, automatic analysis of SD-OCT microstructural features by new computer algorithms may be an effective method to solve such problems.

In this research, we analyzed the features of HRF in different retinal layers. Previous studies indicated the HRF in different retinal layers correlated with different prognosis and therapeutic effect [24, 38]. In the outer retinal layer, the number of HRF at baseline is believed to predict poorer final visual acuity after treatment [24, 38]. In the inner retinal layers, Schreur et al. found that HRF were better responsive to anti-VEGF treatment[19]. In the OPL and ONL where hard exudates mainly distribute within [39], we observed the most HRF, indicating that the HRF are closely associated with hard exudates. Our current result is consistent with the result that the number of HRF correlated with the presence of hard exudate. We believe the current sub-deviation method is helpful for further understanding the pathogenesis of HRF and its clinical significance.

In addition, we observed enhanced signal inside the cysts. Hard exudates are usually considered to be the deposition of macromolecular exudates such as lipids and lipoproteins in retinal tissues after BRB destruction. The enhanced signal in the edema cyst is speculated to be the fibrinogen exudates due to BRB destruction or inflammatory products [40]. In our study, we found that the frequency of hard exudates and enhanced reflectivity in edema cysts in the DME group was significantly higher than that in the other groups, which may indicate that the degree of BRB destruction in the DME group was more severe.

Although the conclusion of this study is that HRF in DME were precursors to hard exudates, we cannot rule out that HRF in other groups of macular edema may represent inflammatory reaction products such as microglia. Some investigators found the number of HRF decrease after anti-inflammatory therapy [36]. We speculated that the reason for the regression of HRF after anti-inflammatory treatment may be that anti-inflammatory treatment improves the BRB and reduces vascular permeability damage; thus, the number of HRF decreases.

The sample size and retrospective nature limited the current study to further analyze the relationship between HRF and more detailed clinical manifestations including the retinal ischemic degree. In the future, prospective studies with large samples representing different causes of macular edema are needed to dynamically observe the changes in HRF to further determine the formation mechanism of HRF.

In conclusion, we demonstrated that HRF in DME patients are closely related to hard exudates. We speculated the HRF in DME patients represent the deposition of large molecular exudates such as proteins and lipids in retinal tissue, which may be the precursor to hard exudates. HRF in DME patients may be an important sign of BRB destruction.

## Data Availability

The datasets used and analyzed during the current study are available from the corresponding author on reasonable request.

## Abbreviations

HRF: Hyperreflective foci
ME: Macular edema
SD-OCT: Spectral-domain optical coherence tomography
VEGF: Vascular endothelial growth factor
DR: Diabetic retinopathy
BRVO: Branch retinal vein occlusion
CRVO: Central retinal vein occlusion
DME: Diabetic macular edema
BRVO-ME: Branch retinal vein occlusion-macular edema
CRVO-ME: Central retinal vein occlusion-macular edema
UME: Uveitic macular edema
EDI: Enhanced depth imaging
ILM: Inner limiting membrane
INL: Inner nuclear layer
MRL: Middle retinal layer
OPL: Outer plexiform layer
ONL: Outer nuclear layer
ORL: Outer retinal layer
ELM: External limiting membrane
RPE: Retinal pigment epithelium
BRB: Blood-retinal barrier
RNFL: Retinal nerve fiber layer
